# Fit (and Healthy) for Duty: Blood Lipid Profiles and Physical Fitness Test Relationships from Police Officers in a Health and Wellness Program

**DOI:** 10.3390/ijerph19095408

**Published:** 2022-04-29

**Authors:** Robert G. Lockie, Robin M. Orr, J. Jay Dawes

**Affiliations:** 1 Department of Kinesiology, California State University, Fullerton, CA 92831, USA; 2 Tactical Research Unit, Bond University, Gold Coast, QLD 4229, Australia; rorr@bond.edu.au; 3 School of Kinesiology, Applied Health and Recreation, Oklahoma State University, Stillwater, OK 74078, USA; jay.dawes@okstate.edu

**Keywords:** aerobic fitness, cardiovascular disease, cholesterol, high-density lipoproteins, law enforcement officer, low-density lipoproteins, police, sit-ups, tactical, triglycerides

## Abstract

This research analyzed archival health and wellness program data (2018: 169 males, 39 females; 2019: 194 males, 43 females) to document police officer lipid profiles, and correlate lipids with fitness. Bloodwork included total cholesterol (TC), low-density lipoproteins (LDL-C), high-density lipoproteins (HDL-C), and triglycerides (TG). Fitness data included maximal aerobic capacity (V^·^O_2max_); sit-and-reach; push-ups; vertical jump; grip strength; sit-ups; and relative bench press (RBP). Lipid profiles were compared to national standards. Spearman’s correlations derived relationships between lipids and fitness (*p* < 0.05). Over 2018–2019, 68–76% of officers had desirable TC (<200 mg/dL) and HDL-C (≥60 mg/dL); 67–72% had desirable TG (<150 mg/dL). 54–62% of officers had LDL-C above desirable (≥100 mg/dL); 13–14% had mildly high TG (150–199 mg/dL); 16–18% had high TG (200–499 mg/dL). In 2018, HDL-C correlated with V^·^O_2max_, push-ups, grip strength, and RBP in males, and sit-ups in females. TG correlated with V^·^O_2max_ (both sexes), sit-ups (males), and grip strength (females). In 2019, TG related to V^·^O_2max_, push-ups, vertical jump, sit-ups, and RBP in males. TG and LDL-C related to push-ups, and HDL-C to sit-ups and RBP in females. Relationship strengths were trivial-to-small (*ρ* = ±0.157 − 0.389). Most officers had good lipid profiles relative to cardiovascular disease risk. Nonetheless, the data highlighted the need for comprehensive approaches to decreasing risk.

## 1. Introduction

Police work can place law enforcement officers at high risk of cardiovascular disease (CVD) [1,2,3]. Some factors that contribute to this risk include stress, shift work, reduced physical activity, decreased sleep time, and poor dietary choices [1,2,3,4]. There is often an interaction between these factors that contribute to poorer health outcomes for police officers. For example, several studies have indicated that the fitness of officers tends to decrease following training academy [5,6,7]. Part of the reason for this is the reduction in physical activity completed by officers once they start working in law enforcement [8,9,10]. Indeed, law enforcement features predominantly sedentary activities (e.g., sitting in a vehicle) during a patrol work shift [4]. Further, shift work can negatively impact the sleep patterns of an officer [11,12], and irregular work hours can contribute to officers making poor food choices [13]. Policing can also impart stress on officers, depending on the tasks they need to perform and their encounters with the general public. For example, police officers may exceed their age-predicted maximum heart rate during certain job tasks (e.g., when driving urgently), which can place greater cardiovascular strain on the officer [14].

Accordingly, it is incumbent that police department command staff encourage their personnel to maintain or potentially improve their health and fitness. Better fitness in law enforcement personnel could contribute to reduced absenteeism and medical care claims [15], decreased risk of injury [16,17,18], and most importantly, improved quality of life for the police officer [19]. One approach that can be utilized are health and wellness programs. These programs are typically multi-faceted with different emphases to cater to the diverse population of law enforcement personnel. These programs can include exercise testing and prescription, nutrition, prevention of injury and chronic disease, interventions for drug use, management of stress, and trauma resilience [20,21,22,23,24]. Health and wellness programs in police departments are generally voluntary; however, certain incentives may be built into the program. As an example, the attainment of certain fitness standards could result in a reward (e.g., financial incentives, paid leave, etc.) for the officer [20,24,25].

In addition to exercise testing (e.g., anaerobic and aerobic fitness tests), health-related assessments can be incorporated into the screening of police officers. Given the propensity for CVD in law enforcement personnel [1,2,3], it is valuable to take metrics that could indicate the risk of this type of disease for the individual. Some examples of these metrics include resting heart rate [26], blood pressure [27], body fat percentage and distribution [28], waist circumference [29,30,31], waist-to-hip ratio [29,30], and blood lipid profiles [32,33,34]. Rodas and Lockie [35] have previously recommended measuring variables such as resting heart rate, blood pressure, and body composition in law enforcement personnel as part of a health and fitness testing battery. Blood lipid profiles could also be beneficial metrics to consider with regards to CVD risk in police officers [3].

Lipid profiles of interest include total cholesterol (TC), low-density lipoproteins (LDL-C), high-density lipoproteins (HDL-C), and triglycerides (TG) [32,33,34]. Cholesterol is a waxy, fat-like substance that is made in the liver and can be found in the blood and cells of the body [36]. Excess cholesterol can build up in blood vessel walls, block blood flow to tissues and organs, and increase CVD risk [36]. High serum levels of LDL-C and TG (fat that can be used for energy) are associated with metabolic syndrome and CVD [37]. HDL-C is referred to as the “good” cholesterol as higher levels can lower the risk of CVD and stroke [32,38,39]. Table 1 details the desired blood lipid levels for TC, LDL-C, HDL-C, and TG in adults [32,33]. This information is in the public domain and has been provided by entities such as the Centers for Disease Control and Prevention [32] and the National Cholesterol Education Program [33], and displayed here for convenience to the reader.

As previously stated, exercise testing and prescription can be a part of police health and wellness programs [20,21,22,23,24]. In a review of literature, Gordon et al. [40] detailed that aerobic conditioning and resistance training can decrease TC, LDL-C, and TG, while increasing HDL-C. Notwithstanding the potential positive impact of exercise, what is also notable is that despite the cardiovascular issues experienced by law enforcement personnel [1,2,3], there is no research that has detailed the lipid profiles from police officers within a health and wellness program. Although there are standards presented for the general population [32,33], it is important that the lipid profiles of police officers are specifically analyzed. It could be expected that fitter officers would be more likely to have better lipid profiles, which would also highlight the importance of fitness (and potentially fitness encouraged by health and wellness program participation) in police officers. However, although these relationships may on the surface appear to be obvious, this ignores the unique challenges and stressors that police officers encounter during their job that negatively impact their health and well-being. Factors such as stress [14,23,41,42,43], disrupted sleep [11,44], dietary challenges [13,22], and decreased physical activity [1,3,4] could all lend themselves to a poorer lipid profile. This may still occur in officers that may still have better physical fitness, as some of these stressors may not be able to be removed from the police officer’s lifestyle (e.g., an officer may always encounter highly stressful environments when encountering offenders from the general population). If officers involved in a health and wellness program demonstrate favorable lipid profiles (Table 1), this would provide further evidence for the value of these types of programs for police departments. Further, no research has detailed relationships between blood lipids and physical fitness in police officers. 

Therefore, the purpose of this study was to detail the lipid profiles of police officers from a health and wellness program in 2018–2019, and correlate lipid profiles with capacities in different fitness tests. It should be reiterated that the purpose of this study was not to track individual police officers across the two years, but rather to analyze the officers as a group to profile blood lipids and correlate these variables with fitness. Indeed, there was variation in officers who participated in fitness testing from year-to-year, so the focus of this research was to investigate data samples as a group from each year (i.e., 2018 and 2019). It was hypothesized that the officers in both years would have good lipid profiles relative to the general population. Additionally, it was hypothesized that there would be significant relationships between a positive blood lipid profile (i.e., lower TC, LDL-C, and TG, and higher HDL-C) and better physical fitness test performance.

## 2. Materials and Methods

### 2.1. Subjects

The data sample provided for this study comprised 447 de-identified officer data sets across two years, including 169 males and 39 females in 2018; and 194 males and 43 females in 2019. The data used in this study has also featured in previously published research [24]. Age and body mass data for both sexes in 2018 and 2019 is reported in Table 1. Height data were not provided to the investigators, but this has happened in previous police research [17,24,45]. All available data were included. Exclusion criterion were data sets with missing data, whether this was due to error or because an officer did not complete a fitness test. Based on the retrospective nature of this analysis, the institutional ethics committee approved the use of pre-existing data (HSR-17-18-370). The study was conducted according to the Declaration of Helsinki [46].

### 2.2. Procedures

The methods for the fitness testing involved in this study have been detailed by Lockie et al. [24]. Nonetheless, they will be briefly detailed here where appropriate. Program participation was voluntary for the officers. Financial incentives could be provided if the officers reached certain milestones within the health and fitness tests. An outsourced wellness provider conducted all testing on-site at the police department. The staff were trained in the required procedures for each test, and police officers were scheduled depending on availability. Testing was on-going throughout 2018 and 2019, and at various times during the day (typically between 8:00 a.m. and 5:00 p.m.). Officers who participated could be coming in for testing during a shift, before or after a shift, or on an off day. This occurred due to the frequent scheduling conflicts that occur for police officers (e.g., shift work, irregular working hours, overtime, court appearances, family and personal commitments, etc.) [47]. Regardless, this research represents real-world outcomes from a police department health and wellness program [24].

Officers began procedures in an office completing the departmental release paperwork. Officers had their age, height (as noted, this height data was not provided to the researchers), body mass, fat mass percentage, and resting blood pressure recorded, and then blood was drawn for the lipid profiling. The police officer then completed the fitness tests in the order presented, unless there was a certain test that the officer did not complete due to physical limitations, such as a pre-existing injury. Within the context of this study, if an officer did not complete a fitness test due to injury, their data were excluded from the analysis. Testing took approximately 60 min to complete.

### 2.3. Lipid Profiles

Bloodwork (TC, LDL-C, HDL-C, TG) was collected in a fasted or non-fasted state at the selection of the officer. Specific details as to which officers had blood drawn when fasted or non-fasted were not provided to the researchers, and indeed it could be considered a limitation that all officers were not measured in the same manner. However, as noted, this was unavoidable considering the job and scheduling demands placed on the officers, and when they were available to participate in the health and wellness program. Some of these demands include long work hours and the impacts of shift work [47]. Furthermore, there are limited clinical differences when comparing fasted or non-fasted lipid profiles [48,49], so this approach was appropriate in the context of this research. Blood was drawn by trained personnel working for the outsourced wellness provider using standard procedures [48,50]. Similar to previous research, the blood samples were processed by an external laboratory to document the concentrations of TC, LDL-C, HDL-C, and TG [50]. As has been done in the literature, lipid concentrations were determined via serum [31,51,52]. The details for the tests were provided by the laboratory on their website [53]. Briefly, as detailed by Labcorp [53], the test used within the laboratory was for the direct determination of LDL-C in nonfasting patients or in patients whose fasting triglycerides were >400 milligrams per deciliter of blood (mg/dL). Laboratory estimation of LDL-C was limited to fasting samples with triglycerides <400 mg/dL [53], and was most commonly determined by the use of formulas such as the Friedewald formula [54]. As noted, more details regarding the lipid testing were provided by Labcorp [53], and the reader is directed to this site for more information. Once the laboratory processed the samples, the data were then sent back to the outsourced wellness provider. All lipids were measured in mg/dL.

### 2.4. Estimated Maximal Aerobic Capacity (V^·^O_2max_)

The officers had their estimated maximal aerobic capacity (V^·^O_2max_) measured via the Gerkin submaximal treadmill testing protocol. This protocol has been used for police officers [24] and firefighters [55,56]. Briefly, heart rate was monitored via a 12-lead electrocardiogram. The test began with a 3-min warm-up at 4.83 km per h [km/h] [55,56]. Following this, treadmill speed was increased to 7.2 km/h. Speed (0.8 km/h) and grade (2%) were alternately increased every 60 s (s) until a heart rate above 85% age-predicted maximum heart rate was achieved. Each 60-s stage consisted of four 15-s intervals, and time to 85% age-predicted maximum heart rate was recorded as the 15-s interval before the achievement of this heart rate [56]. Time was used in a prediction equation within internal documentation from the wellness provider to calculate estimated V^·^O_2max_ in milliliters per kilogram per minute (mL/kg/min) [24]. The formula used has been documented previously in the literature: V^·^O_2max_ = 1.39 (VO_2_ at 85% of age-predicted maximum heart rate) [55].

### 2.5. Sit-and-Reach

The sit-and-reach test measured hamstring flexibility [57], via the use of a sit-and-reach box (Novel Products, Inc., Rockton, IL, USA). The health and wellness staff utilized established protocols covered in detail within the literature [24,57,58], and the reader is directed to these studies. Three trials were performed for the sit-and-reach, with the furthest reach distance measured in centimeters (cm) recorded by staff.

### 2.6. Push-Ups

Upper-body muscular endurance was assessed via a maximal push-up test where the officer completed as many consecutive repetitions as possible without time restrictions [24,59]. A block with a diameter of approximately 6 cm was placed under the chest of the officer to ensure the correct depth was reached [60,61]. The standard push-up technique was used to complete each repetition, which has been detailed in numerous studies [24,60,61,62]. Officers performed as many push-ups as possible until failure or there was a pause identified in the repetition cadence by the staff.

### 2.7. Vertical Jump (VJ)

A VJ, with jump height measured with a Vertec apparatus (Perform Better, West Warwick, RI, USA), was used to infer lower-body power. Established protocols were used, and these have been detailed in previous studies [24,63,64,65]. Officers completed three trials, with the best trial used and jump height measured in inches. The researchers then converted the VJ height to cm for this study.

### 2.8. Grip Strength

Grip strength was measured by a hand grip dynamometer in kg (Takei Scientific Instruments, Niigata City, Japan). This test provides a measure of upper-body strength [66], and previously detailed procedures were used for this test [24,67,68]. Two trials were completed for each hand [24,68]. The best scores for the left and right hands were added together to present a combined score. 

### 2.9. Sit-Ups

Abdominal muscular endurance was assessed via the 60-s sit-up test. The procedures for this test have been described in various studies [7,24,58,61]. As part of the health and wellness program, officers could complete a plank instead of sit-ups if they had lower back issues. Plank data were not included in this research.

### 2.10. Bench Press

Absolute and relative upper-body strength was measured by the 1RM bench press. Established protocols were used by the staff [24,69], but the methods will be briefly stated here. A Smith machine, bench, and weight plates were used. The officer laid on the bench (head, shoulders, and buttocks in contact with the bench) with their feet flat on the floor. The officer began the lift with their arms extended and gripping the bar with a pronated grip. To complete a repetition, officers were to allow the bar to touch the chest, pause briefly, and then push the bar to full elbow extension. Officers with shoulder issues were provided the option to pause at a positon of approximately 90° elbow flexion. Lifting procedures (i.e., loading used, rest periods between attempts, number of attempts, what constituted a failed repetition) have been detailed by Lockie et al. [24]. Absolute strength was the maximum bench press load lifted once [24,69]. Relative strength was derived by the following formula: 1RM/body mass [24,69].

### 2.11. Statistical Analysis

Statistical analyses were processed using the Statistics Package for Social Sciences (Version 27; IBM Corporation, New York, NY, USA). Descriptive statistics (mean ± standard deviation (SD)) were calculated for each metric. As the data represented actual police officers from the field, it was important to include all available and complete datasets in the current analysis. In order to categorize the police officer’s lipid profiles, data were analyzed by year with TC, LDC, HDC, and TG compared to national standards [32,33]. Preceding the correlation analysis, normality of the data was evaluated by visual analysis of Q-Q plots [70,71,72] and the Kolmogorov–Smirnov test [73]. As will be detailed in the Results section, most of test data for the officers in each year were determined to be not normally distributed. This meant that Spearman’s correlations were utilized to calculate relationships between blood lipids and the fitness tests. Spearman’s correlations were used as they are more robust and appropriate for non-parametric data [74,75]. The sexes were analyzed separately as numerous studies have documented differences between males and female law enforcement personnel in physical performance tests [60,61,63,67,76]. Significance was set at *p* < 0.05 a priori. The strength of the correlations (*ρ*) was defined as: a *ρ* between 0 to ±0.3 was small; ±0.31 to ±0.49, moderate; ±0.5 to ±0.69, large; ±0.7 to ±0.89, very large; and ±0.9 to ±1, near perfect for predicting relationships [77].

## 3. Results

**Figure 1:**
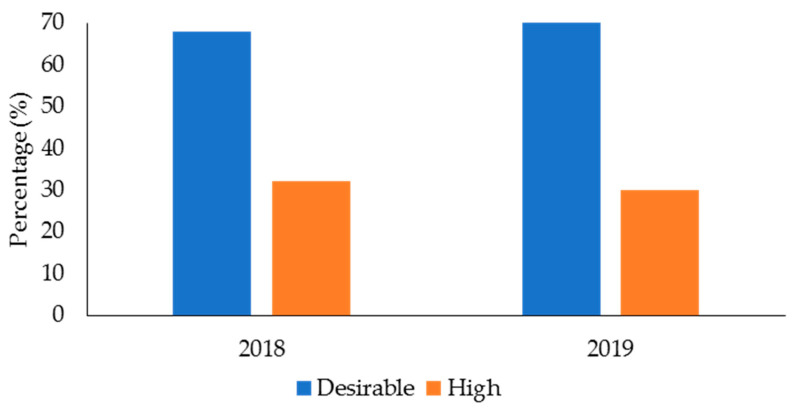
Percentage of police officers from a health and wellness program in 2018 and 2019 classified according to the categories for total cholesterol.

**Figure 2:**
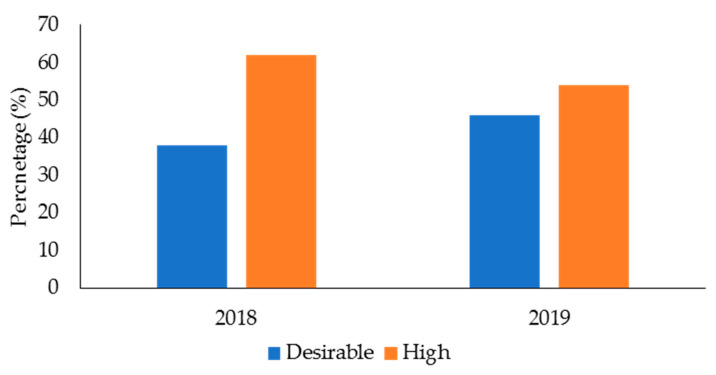
Percentage of police officers from a health and wellness program in 2018 and 2019 classified according to the categories for low-density lipoproteins.

**Figure 3:**
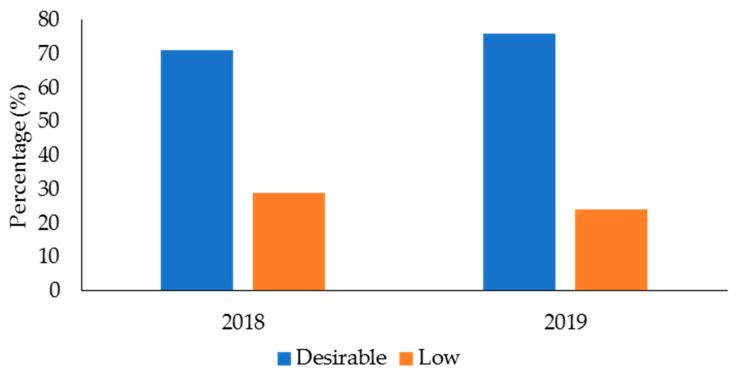
Percentage of police officers from a health and wellness program in 2018 and 2019 classified according to the categories for high-density lipoproteins.

**Figure 4:**
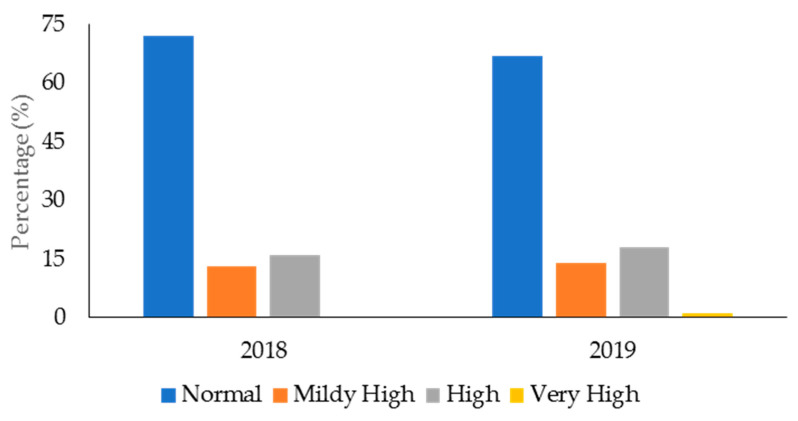
Percentage of police officers from a health and wellness program in 2018 and 2019 classified according to the categories for triglycerides.

The descriptive data for age, body mass, and performance in the fitness tests for the male and female police officers from 2018 and 2019 in the health and wellness program is displayed in Table 2. Table 3 displays the mean data for TC, LDC, HDC, and TG for 2018 and 2019. In 2018 (Figure 1), 140 officers (68% of the sample in this year) had desirable TC, while 66 officers (32%) had high TC. The numbers for TC were similar in 2019 (166 officers (70%) had desirable TC, 71 officers (30%) had high TC). For LDL-C (Figure 2), 78 officers (38%) in 2018 and 106 officers (46%) in 2019 had desirable levels; 127 officers (62%) in 2018 and 125 officers (54%) had high LDL-C levels. With regards to HDL-C (Figure 3), 147 officers (71%) had desirable levels in 2018, while 179 officers (76%) had desirable levels in 2019. There were 59 officers (29%) in 2018, and 58 officers (24%) in 2019, who had low HDL-C levels. Regarding TG (Figure 4), in 2018, 148 officers (72%) had normal levels, 26 (13%) were mildly high, and 32 (16%) were high. In 2019, 160 officers (67%) had normal TG levels, 33 (14%) were mildly high, 42 (18%) were high, and 3 (1%) were very high.

According to the Kolmogorov–Smirnov tests for the 2018 data, 10 of 12 variables were significant and deemed to have non-normal distribution (*p* ≤ 0.047). In 2019, eight of 12 variables had non-normal distribution (*p* ≤ 0.026). Accordingly, as most variables in each year were not normally distributed, Spearman’s correlations were utilized in this study. The correlation data for the male and female police officers in 2018 is shown in Table 4 and Table 5, respectively. For male officers (Table 4), there were significant positive correlations between HDL-C with V^·^O_2max_, push-ups, grip strength, and relative bench press (all trivial effects). For female officers (Table 5), there was a significant, small relationship between HDL-C with sit-ups. These relationships suggested that a higher HDL-C level was associated with a higher V^·^O_2max_, more push-up repetitions, and greater grip strength and relative bench press in males, and more sit-up repetitions in females. There were significant, negative correlations between TG and V^·^O_2max_ in male (trivial) and female (small) officers, sit-ups in males (trivial), and grip strength in females (small). These relationships suggested that lower TG levels were associated with a higher V^·^O_2max_, more sit-up repetitions, or greater grip strength. There were no other significant correlations between lipid levels and fitness test performance in 2018 for male or female police officers.

The correlation data for the male and female police officers in 2019 is shown in Table 6 and Table 7, respectively. For male officers (Table 6), there were no significant correlations between TC, LDL-C, or HDL-C with any fitness test. There were significant, negative correlations between TG with estimated V^·^O_2max_, push-ups, vertical jump, sit-ups, and relative bench press (all trivial effects). The relationships indicated that lower TG was associated with better performance in each fitness test. For female officers (Table 7), there were small, positive relationships between TG and LDL-C with push-ups. These relationships suggested that higher TG and LDL-C was associated with more push-up repetitions. There were also small, positive relationships between HDL-C with sit-ups and relative bench press; higher HDL-C was related to more sit-up repetitions and a greater relative bench press. There were no other significant associations between lipids and fitness test performance in 2019 female officers.

## 4. Discussion

This study described the lipid profiles of police officers within a health and wellness program in 2018 and 2019. Further to this, the relationships between blood lipids and different measures of physical fitness were also profiled. Firstly, it is important to note that most officers participating in the health and wellness program had good lipid profiles relative to CVD risk. While this is a positive result, these results could also be influenced by the healthy worker effect, which is a bias that can occur in occupational epidemiology studies [78,79]. In the context of this study, this would mean that less healthy officers were more likely to not participate in the health and wellness program. Potentially, the healthier police officers in the department would be involved with the program and may provide the impression that the police department is healthier than they might otherwise be. Nevertheless, command staff from the police department should view the data from this study as a positive outcome relative to the general health of their participating officers and note the value of such programs. This is ultimately a goal of these types of programs for police departments [20,21,22,23,24]. Furthermore, as will be discussed, there were officers who had poorer lipid profiles who would benefit from continued participation in the health and wellness program.

Ideally, police officers should possess healthy TC, LDL-C, and TG levels. Excess TC, LDL-C, and TG can lead to fatty deposits in the arteries, which can cause atherosclerosis [80,81]. Atherosclerosis is the thickening or hardening of the arteries caused by a buildup of plaque in the inner lining of an artery and is the major underlying cause of CVD [82]. The results from this study showed that most police officers from the health and wellness program (68–70% across the two years) had desirable TC levels. This was also true for TG, with 67–72% of officers having desirable levels. However, 54–62% of police officers from the health and wellness program had high LDL-C. Additionally, 13–14% of officers had mildly/borderline high TG, and 16–19% had high-to-very high TG. 

Numerous genetic and environmental factors can influence the onset of atherosclerosis [82]. The nature of police work (i.e., decreased physical activity, shift work, stress, decreased sleep, poor diet) [1,2,3,4] likely contributed to these less favorable blood lipid profiles in some of the officers from this study. These data indicate the need for specific interventions to improve the health and potentially quality of life for police officers that have unfavorable blood lipid profiles. For example, regular exercise [83], dietary improvements [84], and elimination of behaviors such as smoking [52] and excessive alcohol intake [85] can help lower TC, LDL-C, and TG. Health and wellness programs for police departments commonly seek to address these issues for their personnel [20,21,22,23,24], and multifaceted approaches could be the best approach to enhancing the health and fitness of police officers [22]. It should be noted that although dietary interventions can be beneficial for improving factors that relate to CVD risk (e.g., blood lipid profiles, weight management) [39,86,87], these interventions should be made within the context of occupational challenges and motivations experienced by police officers [13]. This is also true for physical activity [8,88]. Indeed, the potential benefit of health and wellness programs designed for police departments is that they can cater to specific needs and constraints of officers. Longitudinal research should be conducted in the future to ascertain the best practices for encouraging program participation and health and fitness changes in police officers.

HDL-C are considered the ‘good cholesterol’ [32], and can lower the risk of CVD and stroke [38,39]. HDL-C promotes cholesterol efflux and reverse cholesterol transport, in addition to modulating inflammation [38]. Most of the officers involved in this health and wellness program had desirable HDL-C levels. Even though these data could be influenced by the healthy worker effect [78,79], it is still a positive outcome for the health and wellness program that most of the participants had desirable HDL-C levels. Nonetheless, it is important to note the 29% of officers in the sample from 2018, and 24% of officers in 2019, showed low HDL-C levels. These officers would benefit from some type of intervention to help boost their HDL-C levels and reduce their risk of CVD. Similar to the recommendations for improving TC, LDC, and TG profiles, regular exercise, dietary interventions (e.g., lower saturated fat, caloric restriction), weight reduction if overweight or obese, and eliminating smoking can help increase HDL-C levels [39,87,89,90]. Effective health and wellness programs could address these concerns for officers [22], especially those at greater risk of CVD as evidenced by their HDL-C. Future research should investigate the effectiveness of different interventions (e.g., exercise, dietary, or both) as part of a health and wellness program to improving the blood lipid profile and lessening CVD risk in police officers.

There were relatively few significant relationships between blood lipids and fitness test performance, although the significant correlations are worth noting. In 2018, greater HDL-C related to a higher estimated V^·^O_2max_, more push-up repetitions, greater strength measured by grip strength and the relative bench press in male officers, and more sit-up repetitions in female officers. Lower TG related to a higher estimated V^·^O_2max_ in male and female officers, more sit-up repetitions in male officers, and greater grip strength in female officers. In 2019, lower HDL-C related to greater sit-up repetitions and a higher relative bench press in female officers. In male officers, lower TG related to a higher estimated V^·^O_2max_, more push-up and sit-up repetitions, and a greater vertical jump and relative bench press. All these relationships were desirable. However, in 2019, a higher TC and LDL-C were related to more push-ups in female officers, which is less desirable. These data could be related to body size; physically larger females may have greater adiposity which could have contributed to higher TC and LDL-C [31], but more muscle mass which benefits push-up performance [65]. Furthermore, there were certain relationships that were significant in 2018 but not in 2019 (and vice-versa), which may be in part due to the variations in officer participation from one year to the next.

Police officers involved in health and wellness programs tend to complete more physical activity [20], and more physically active law enforcement personnel tend to have better body composition (i.e., greater lean body mass and less fat mass) [91] and reduced incidence of CVD [19]. Day et al. [86] found that nutrition and physical activity programs can also lead to positive physical changes (i.e., weight loss) over six months in firefighters. It could be expected that long-term involvement in health and wellness programs could also lead to positive health outcomes in police officers, although this requires further research. Nevertheless, what should be emphasized is that the strength of any significant relationships in this study between blood lipids and fitness test performance were only trivial-to-small. In addition, there were no other significant correlations between blood lipids and the fitness tests other than those previously stated. The results from the correlation analysis in the current research suggest fitness should be considered somewhat independent of CVD risk as indicated by blood lipid levels. There may be police officers who are physically fit but may not be healthy (i.e., they may still have unfavorable blood lipid profiles). As an example, the positive correlations (albeit small) between TC and LDL-C and push-ups in the 2019 females provides some indication of officers who may have better fitness as measured by a push-up test, but less than ideal blood lipid profiles. The data from this study emphasize the need for a multifaceted approach for wellness programs in order to reduce CVD risk and enhance physical fitness in police officers (i.e., health and fitness testing, exercise programs, dietary interventions, wellness and drug education). 

There are particular limitations to this research study that should be described. Blood lipids were either measured in fasted or non-fasted states depending on the preference of the police officer. This could have affected the results from this study [48]. This limitation is an example of the real-world challenges associated with collecting physiological data from incumbent police officers. Nevertheless, and as previously stated, there are limited clinical differences when measuring blood lipids in fasted or non-fasted states [48,49], so the results from this study still hold great value. The real-world challenges also influenced when officers reported for fitness testing, which could have occurred when officers were coming in on shift, on an off day, or before or after a shift. While it would be ideal to have officers report at consistent times (e.g., on an off day), this was not possible within this department. Indeed, police officers and command staff experience long hours, shift work, and staffing challenges [92]; this can make any form of structured exercise testing challenging. The Gerkin protocol, which was employed to gauge aerobic fitness, may overestimate V^·^O_2max_ in apparently healthy men and women [55]. This was a cross-sectional study, and future research should involve longitudinal analyses of health and wellness programs that are instituted within police departments. Regardless, within the contextual framework of these limitations, the results provided valuable information regarding the blood lipid profiles and CVD risk of police officers participating in a health and wellness program. Further, the limited relationships between blood lipids and physical fitness highlight the need for a multifaceted approach to enhancing the overall health, fitness, and general well-being of police officers.

## 5. Conclusions

Most police officers participating in the health and wellness program from this sample displayed good lipid profiles relative to their risk of CVD. As the health and wellness program involved voluntary (although incentivized) participation, the results may be related to the healthy worker effect. Nevertheless, the police department should view the data from this study as a positive outcome relative to the general health of their participating officers. There were officers who had poorer lipid profiles who would benefit from continued participation in the program, highlighting the significance of such programming within police departments. There were relatively few significant correlations between blood lipids and the physical fitness tests, and the strength of the desirable relationships (HDL-C with estimated V^·^O_2max_, push-ups, grip strength, sit-ups, and relative bench press; TG with estimated V^·^O_2max_, push-ups, vertical jump, grip strength, sit-ups, and relative bench press) tended to be trivial-to-small. These results would seem to indicate that physical fitness should be considered independent of CVD risk as indicated by blood lipid levels (i.e., there could be police officers who are fit but still have unfavorable blood lipid profiles). These data would imply that health and wellness programs should incorporate multiple approaches (e.g., exercise testing and programming, dietary interventions, wellness and drug education, etc.) to reduce CVD risk, enhance physical fitness, and improve quality of life for police officers.

## Figures and Tables

**Table 1:** Desirable blood lipid levels for total cholesterol (TC), low-density lipoproteins (LDL-C), high-density lipoproteins (HDL-C), and triglycerides (TG) [32,33]. All lipid levels are measured in milligrams per deciliter (mg/dL).

Lipid	Levels
TC	<200 mg/dL
LDL-C	<100 mg/dL
HDL-C	≥60 mg/dL
TG	<150 mg/dL

**Table 2:** Descriptive data (mean ± SD) for age, body mass, and fitness test performance (estimated maximal aerobic capacity (V^·^O_2max_), sit-and-reach, push-ups, vertical jump, grip strength, sit-ups, one-repetition maximum (1RM) bench press and relative bench press ratio) for male and female police officers from health and wellness programs in 2018 and 2019.

Age, Body Mass, and Fitness Tests	2018		2019	
	Males (*n* = 169)	Females (*n* = 39)	Males (*n* = 194)	Females (*n* = 43)
Age (years)	40.4 ± 8.1	35.3 ± 9.2	39.6 ± 8.4	35.4 ± 8.8
Body Mass (kg)	90.52 ± 15.50	69.45 ± 14.14	89.08 ± 13.08	70.16 ± 17.00
Estimated V^·^O_2max_ (mL/kg/min)	55.39 ± 8.84	47.42 ± 8.16	55.03 ± 8.74	47.94 ± 7.54
Sit-and-Reach (cm)	29.05 ± 8.56	34.22 ± 8.80	29.39 ± 8.27	34.34 ± 7.51
Push-ups (repetitions)	47.74 ± 17.27	27.58 ± 13.70	49.64 ± 17.47	28.47 ± 10.77
Vertical Jump (cm)	60.88 ± 9.88	39.54 ± 7.93	60.67 ± 8.76	40.86 ± 6.36
Grip Strength (kg)	98.88 ± 14.99	58.67 ± 9.09	102.26 ± 16.25	60.48 ± 9.35
Sit-ups (repetitions)	43.47 ± 10.55	32.09 ± 12.42	45.67 ± 8.80	42.63 ± 57.78
1RM Bench Press (kg)	107.84 ± 22.34	49.71 ± 10.83	104.34 ± 25.33	51.50 ± 11.41
Relative Bench Press (kg/body mass)	1.21 ± 0.26	0.74 ± 0.19	1.19 ± 0.28	0.73 ± 0.17

**Table 3:** Descriptive data (mean ± SD) for total cholesterol (TC), low-density lipoproteins (LDL-C), high-density lipoproteins (HDL-C), and triglycerides (TG) in male and female police officers from health and wellness programs in 2018 and 2019. All lipid levels are measured in milligrams per deciliter (mg/dL).

Lipids	2018		2019	
	Males (*n* = 170)	Females (*n* = 39)	Males (*n* = 194)	Females (*n* = 44)
TC (mg/dL)	188.39 ± 41.93	177.32 ± 27.36	189.24 ± 43.52	177.00 ± 32.38
LDL-C (mg/dL)	114.74 ± 34.11	100.65 ± 27.46	108.30 ± 32.60	94.42 ± 26.20
HDL-C (mg/dL)	51.96 ± 13.17	59.79 ± 14.06	50.58 ± 13.67	59.72 ± 13.53
TG (mg/dL)	140.78 ± 89.80	116.76 ± 73.86	150.49 ± 104.95	114.23 ± 58.90

**Table 4:** Relationships between blood lipids (total cholesterol (TC), low-density lipoproteins (LDL-C), high-density lipoproteins (HDL-C), and triglycerides (TG)) and fitness (estimated maximal aerobic capacity (V^·^O_2max_), sit-and-reach, push-ups, vertical jump, grip strength, sit-ups, one-repetition maximum (1RM) bench press, and relative bench press) in male police officers (*n* = 169) from a health and wellness program in 2018. Significant relationships are highlighted in orange.

		TC	LDL-C	HDL-C	TG
Estimated V^·^O_2max_	*ρ*	−0.048	−0.118	0.291 *	−0.158 *
	*p*	0.542	0.129	<0.001	0.041
Sit-and-Reach	*ρ*	0.046	0.046	0.112	−0.108
	*p*	0.556	0.559	0.149	0.163
Push-ups	*ρ*	0.096	0.079	0.175 *	−0.105
	*p*	0.216	0.310	0.023	0.175
Vertical Jump	*ρ*	−0.079	−0.094	0.083	−0.087
	*p*	0.314	0.233	0.291	0.266
Grip Strength	*ρ*	−0.004	−0.037	0.169 *	−0.121
	*p*	0.963	0.636	0.028	0.118
Sit-ups	*ρ*	−0.041	−0.066	0.138	−0.233 *
	*p*	0.613	0.415	0.089	0.004
1RM Bench Press	*ρ*	−0.141	−0.102	0.016	−0.112
	*p*	0.069	0.190	0.839	0.149
Relative Bench Press	*ρ*	−0.024	−0.013	0.157 *	−0.144
	*p*	0.762	0.872	0.042	0.063

* Significant (*p* < 0.05) relationship between the two variables.

**Table 5:** Relationships between blood lipids (total cholesterol (TC), low-density lipoproteins (LDL-C), high-density lipoproteins (HDL-C), and triglycerides (TG)) and fitness (estimated maximal aerobic capacity (V^·^O_2max_), sit-and-reach, push-ups, vertical jump, grip strength, sit-ups, one-repetition maximum (1RM) bench press, and relative bench press) in female police officers (*n* = 39) from a health and wellness program in 2018. Significant relationships are highlighted in orange.

		TC	LDL-C	HDL-C	TG
Estimated V^·^O_2max_	*ρ*	−0.134	−0.079	0.163	−0.343 *
	*p*	0.423	0.642	0.328	0.035
Sit-and-Reach	*ρ*	−0.226	−0.137	−0.005	0.065
	*p*	0.172	0.419	0.974	0.700
Push-ups	*ρ*	−0.091	−0.023	0.098	−0.151
	*p*	0.592	0.894	0.565	0.372
Vertical Jump	*ρ*	−0.202	−0.164	0.156	−0.176
	*p*	0.231	0.341	0.356	0.297
Grip Strength	*ρ*	0.079	0.008	0.300	−0.380 *
	*p*	0.636	0.963	0.067	0.019
Sit-ups	*ρ*	−0.014	−0.105	0.380 *	−0.200
	*p*	0.937	0.568	0.029	0.265
1RM Bench Press	*ρ*	−0.011	0.033	0.098	−0.115
	*p*	0.947	0.846	0.560	0.491
Relative Bench Press	*ρ*	−0.211	−0.125	0.126	−0.277
	*p*	0.204	0.460	0.452	0.093

* Significant (*p* < 0.05) relationship between the two variables.

**Table 6:** Relationships between blood lipids (total cholesterol (TC), low-density lipoproteins (LDL-C), high-density lipoproteins (HDL-C), and triglycerides (TG)) and fitness (estimated maximal aerobic capacity (V^·^O_2max_), sit-and-reach, push-ups, vertical jump, grip strength, sit-ups, one-repetition maximum (1RM) bench press, and relative bench press) in male police officers (*n* = 194) from a health and wellness program in 2019. Significant relationships are highlighted in orange.

		TC	LDL-C	HDL-C	TG
Estimated V^·^O_2max_	*ρ*	−0.062	−0.054	0.118	−0.163 *
	*p*	0.396	0.465	0.107	0.026
Sit-and-Reach	*ρ*	0.048	0.066	0.141	−0.083
	*p*	0.505	0.372	0.050	0.254
Push-ups	*ρ*	−0.063	0.032	0.065	−0.275 *
	*p*	0.383	0.666	0.367	<0.001
Vertical Jump	*ρ*	−0.124	−0.046	−0.006	−0.163 *
	*p*	0.087	0.533	0.930	0.025
Grip Strength	*ρ*	0.031	0.094	−0.001	−0.111
	*p*	0.668	0.199	0.985	0.123
Sit-ups	*ρ*	−0.113	−0.151	0.155	−0.222 *
	*p*	0.157	0.059	0.051	0.005
1RM Bench Press	*ρ*	−0.057	0.018	−0.092	−0.111
	*p*	0.426	0.808	0.203	0.122
Relative Bench Press	*ρ*	−0.027	0.046	0.061	−0.188 *
	*p*	0.711	0.530	0.401	0.009

* Significant (*p* < 0.05) relationship between the two variables.

**Table 7:** Relationships between blood lipids (total cholesterol (TC), low-density lipoproteins (LDL-C), high-density lipoproteins (HDL-C), and triglycerides (TG)) and fitness (estimated maximal aerobic capacity (V^·^O_2max_), sit-and-reach, push-ups, vertical jump, grip strength, sit-ups, one-repetition maximum (1RM) bench press, and relative bench press) in female police officers (*n* = 43) from a health and wellness program in 2019. Significant relationships are highlighted in orange.

		TC	LDL-C	HDL-C	TG
Estimated V^·^O_2max_	*ρ*	−0.004	0.093	−0.142	0.036
	*p*	0.978	0.559	0.369	0.822
Sit-and-Reach	*ρ*	−0.218	−0.280	−0.023	−0.115
	*p*	0.160	0.069	0.884	0.463
Push-ups	*ρ*	0.389 *	0.335 *	0.001	0.274
	*p*	0.013	0.035	0.996	0.088
Vertical Jump	*ρ*	0.179	0.117	0.067	0.075
	*p*	0.264	0.466	0.679	0.639
Grip Strength	*ρ*	0.261	0.212	0.071	0.198
	*p*	0.092	0.173	0.653	0.204
Sit-ups	*ρ*	0.030	−0.082	0.364 *	−0.220
	*p*	0.872	0.655	0.040	0.227
1RM Bench Press	*ρ*	0.098	−0.002	0.150	0.086
	*p*	0.533	0.988	0.336	0.581
Relative Bench Press	*ρ*	0.096	−0.075	0.312 *	−0.044
	*p*	0.544	0.635	0.044	0.781

* Significant (*p* < 0.05) relationship between the two variables.

## Data Availability

Restrictions apply to the availability of these data due to ethical, legal and privacy concerns.
